# Tacrolimus Inhibits the Revascularization of Isolated Pancreatic Islets

**DOI:** 10.1371/journal.pone.0056799

**Published:** 2013-04-17

**Authors:** Ryuichi Nishimura, Sho Nishioka, Ikuma Fujisawa, Hitoshi Shiku, Miki Shimada, Satoshi Sekiguchi, Keisei Fujimori, Akira Ushiyama, Tomokazu Matsue, Noriaki Ohuchi, Susumu Satomi, Masafumi Goto

**Affiliations:** 1 Division of Advanced Surgical Science and Technology, Tohoku University, Sendai, Japan; 2 Graduate School of Environmental Studies, Tohoku University, Sendai, Japan; 3 Department of Pharmaceutical Sciences, Tohoku University Hospital, Sendai, Japan; 4 Department of Environmental Health, National Institute of Public Health, Wako, Japan; 5 WorldPremier InternationalResearch Center Initiative Advanced Institute for Materials Research, Tohoku University, Sendai, Japan; 6 New Industry Creation Hatchery Center, Tohoku University, Sendai, Japan; University of Bremen, Germany

## Abstract

**Aims:**

Immunosuppressive drugs could be crucial factors for a poor outcome after islet allotransplantation. Unlike rapamycin, the effects of tacrolimus, the current standard immunosuppressant used in islet transplantation, on graft revascularization remain unclear. We examined the effects of tacrolimus on islet revascularization using a highly sensitive imaging system, and analyzed the gene expression in transplanted islets by introducing laser microdissection techniques.

**Methods:**

Islets isolated from C57BL/6-Tg (CAG-EGFP) mice were transplanted into the nonmetallic dorsal skinfold chamber on the recipients. Balb/c athymic mice were used as recipients and were divided into two groups: including a control group (n = 9) and tacrolimus-treated group (n = 7). The changes in the newly-formed vessels surrounding the islet grafts were imaged and semi-quantified using multi-photon laser-scanning microscopy and a Volocity system. Gene expression in transplanted islets was analyzed by the BioMark dynamic system.

**Results:**

The revascularization process was completed within 14 days after pancreatic islet transplantation at subcutaneous sites. The newly-formed vascular volume surrounding the transplanted islets in the tacrolimus-treated group was significantly less than that in the control group (p<0.05). Although the expression of *Vegfa* (p<0.05) and *Ccnd1* (p<0.05) was significantly upregulated in the tacrolimus-treated group compared with that of the control group, no differences were observed between the groups in terms of other types of gene expression.

**Conclusions:**

The present study demonstrates that tacrolimus inhibits the revascularization of isolated pancreatic islets without affecting the characteristics of the transplanted grafts. Further refinements of this immunosuppressive regimen, especially regarding the revascularization of islet grafts, could improve the outcome of islet allotransplantation.

## Introduction

Pancreatic islet transplantation is an emerging and promising therapy for type 1 diabetes. However, at least two donor pancreata are needed to reverse hyperglycemia, and substantial number of islet grafts fail within 5 years [1]. Although several factors may contribute to a progressive decline in the function of islet transplants, the main reason for this problem is still uncertain. Pancreatic islet autotransplantation, despite the use of a lower beta-cell mass, shows good results and improves insulin-independence rates in comparison to the islet allotransplantation [2]. Pancreatic islet allografts are affected by various factors, including relatively long cold ischemia times before pancreatic islet isolation [3], [4], the need for a purification procedure, and the use of immunosuppressive drugs to regulate the alloimmune responses to the islet grafts.

The inhibitory effects of rapamycin, a key component of the immunosuppressive regimen in the Edmonton protocol, on tumor angiogenesis or pancreatic islet revascularization have recently been clarified [5], [6], [7], 8,9. However, the inhibitory effects of tacrolimus, which is one of the standard immunosuppressants in both pancreatic islet transplantation and whole pancreas transplantation, on revascularization has been poorly studied, although Turgut et al. reported the prevention of corneal neovascularization by the systemic and topical administration of tacrolimus [10].

Therefore, this study sought to evaluate the effect of tacrolimus on the islet revascularization process using a highly sensitive imaging system combined with the dorsal skinfold chamber (DSC) technique and multi-photon laser-scanning microscopy (MPLSM). This new system enabled repeated observation of the time-dependent changes of islet grafts and surrounding vessels in identical animals without histological sectioning [11]. Spier et al. introduced the anterior chamber of the eye for monitoring revascularization of the islet grafts by using MPLSM [12], [13]. Although the anterior chamber of the eye is a breakthrough model, it is not realistic for clinical application. In contrast, the subcutaneous space is an ideal site for islet transplantation due to the minimal invasion and easy access. The liver, which is the current standard transplant site, is often associated with procedure-related complications including hemorrhage, thrombosis, and the strong innate immune response such as the instant blood-mediated inflammatory reaction (IBMIR) [14], [15], thus the current model could be a useful and practical tool to analyze the revascularization process of islet grafts.

Hence, the present study examined the effect of tacrolimus on islet revascularization using a highly sensitive imaging system. Furthermore, this study attempted to analyze the state of gene expression in transplanted islet grafts *per se* by introducing laser microdissection techniques.

## Materials and Methods

### Mouse models

All animals used in this study were handled in accordance with the Guide for the Care and Use of Laboratory Animals published by the National Institutes of Health [16]. The animal studies were approved by the Animal Care and Use Committee of the Tohoku University (approved protocol ID: 2011 NICHe-Animal-13). All surgeries were performed under anesthesia, and all efforts were made to minimize suffering. Male C57BL/6-Tg (CAG-EGFP) mice (9–12 weeks of age; Japan SLC Inc., Shizuoka, Japan) were used as islet donors.

**Figure 1:**
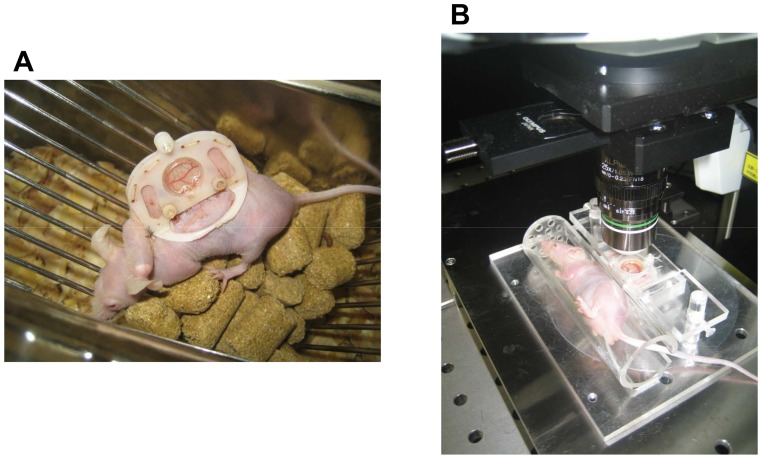
Mice bearing the dorsal skinfold chamber (A). Mice during microscopic observation (B). The mice were positioned in an acrylic resin tube during microscopic observation. The tube was fixed on an acrylic resin plate and then placed on the microscope stage.

The dorsal skinfold chamber (DSC), which is composed of polyacetal resin (generally known as Duracon; grade M90-44, Polyplastics Co.,Ltd., Tokyo, Japan) [17], was introduced into the recipient mice [11] (Fig. 1A). This nonmetallic chamber is approximately 40% lighter in weight than metallic chamber used traditionally, subjecting mice to less stress. DSC was implanted into male Balb/c nu/nu mice (8–12 weeks of age; Japan SLC Inc.). This model, unlike wild type Balb/c mice, allowed observation for a relatively long preservation period and also to focus on the influence of immunosuppressive chemicals on engraftment without any interference from the specific immune responses.

All animals were bred and maintained in a pathogen-free environment with free access to laboratory chow and water and exhibited no signs of discomfort.

### Pancreatic islet isolation and culture

C57BL/6-Tg (CAG-EGFP) mice were anesthetized with the inhalation of isoflurane (Abbott Japan Co., Ltd., Tokyo, Japan). The bile duct was identified and clamped at the papilla Vateri. Four milliliters of cold Hank's balanced salt solution (HBSS) containing 1 g/L collagenase (Sigma type V; Sigma Chemicals, St. Louis, MO, USA) was injected into the common bile duct leading to the pancreas under a stereomicroscope. The pancreas was removed and incubated in a water bath at 37°C for 16 min. The pancreas was digested, the cell suspension was washed two times in HBSS and centrifuged for 1 min. Density-gradient centrifugation was performed for 10 min using Histopaque-1119 (Sigma Diagnostics, St. Louis, MO, USA) and Lymphoprep^TM^ (Nycomed Pharma AS, Oslo, Norway) to isolate pancreatic islets. The islets were cultured in RPMI-1640 containing 5.5 mmol/L glucose and 10% fetal bovine serum at 37°C in 5% CO_2_ and humidified air for approximately 3 hours before examination.

### Islet transplantation into the dorsal skinfold chamber

Balb/c nu/nu mice underwent islet transplantation into the DSC under isoflurane (Abbott Japan Co., Ltd.) anesthesia. Two to ten of isolated islets were transplanted into each DSC by means of the hand-picking method. The space between skin flap and the cover glass was filled with saline to remove air bubbles. The recovery rate of the grafts at the end of the observation period was almost same between the tacrolimus and control groups.

### Experimental groups

The recipient mice with transplanted pancreatic islets were divided into two groups: the control group (n = 9) and the tacrolimus (Astellas, Deerfield, IL)-treated group (0.5 mg/kg/day; n = 7, two of seven islets were observed up to 11 days after transplantation due to technical trouble of DSC.) [18]. Tacrolimus was administered to mice via subcutaneous implanted MICRO-OSMOTIC PUMP (Model 1002, Alzet, Cupertino, CA) for 14 days. The control mice were also implanted with a MICRO-OSMOTIC PUMP and treated with vehicle.

### Measurement of tacrolimus concentrations in the whole blood

Blood samples were obtained via the inferior vena cava at 7 days after the initial administration of tacrolimus. Blood samples were stored at −80°C until use. Tacrolimus concentrations in the whole blood were measured by using Dimension TACRO (Siemens Healthcare Diagnostics, Inc., Newark, DE, USA).

### *In vivo* imaging of islets transplanted into the dorsal skinfold chamber

A 0.1 mL sample of Texas Red (10 mg/mL; Invitrogen, Leek, The Netherlands) was injected intravenously via the tail vein. The mice were anesthetized using the inhalation of isoflurane and then were positioned in an acrylic resin tube with an inner diameter of 26 mm. The tube was fixed on an acrylic resin plate and then placed on the microscope stage (Fig. 1B). A multiphoton laser-scanning microscope (MPLSM; FluoView FV1000MPE; OLYMPUS, Tokyo, Japan) equipped with water-dipping lenses (OLYMPUS XLPLN25XWMP NA1.05; OLYMPUS) was used to image the transplanted islet grafts. Distilled water was used as immersion liquid. MPLSM imaging was performed under the minimum required laser-power and scan-time necessary. No signs of photo-damage in islet cells or blood vessels were observed throughout the whole study. I processed images and measured newly-formed vascular volume surrounding the islets using a Volocity 3D system (PerkinElmer, Waltham, MA, USA) to evaluate islet revascularization. The newly-formed vascular volume surrounding the islets at each time point was calculated by the increasing rate against day 1 after transplantation. GFP and Texas Red were excited at 890 nm and separated and collected emission light onto two non-descanned detectors using a dichroic mirror (FV10-MRG/R:DM570) and emission filters (FV10-MRG/R:BA495-540HQ and BA575-630) [12], [13].

### Laser microdissection

The samples were divided into three groups: 1) islets before transplantation(n = 6), 2) islets in the control group(n = 6), 3) islets in the tacrolimus-treated group(n = 6). Tissues containing pancreatic islets, except for pancreatic islets before transplantation, were harvested at 7 days after transplantation, frozen with liquid nitrogen and stored at −80°C until use. Ten µm frozen sections were prepared from each of these samples. Frozen prepared sections were fixed with acetone for 30 seconds, and then dehydrated and air dried immediately before performing laser microdissection (LMD). LMD was performed using LMD 7000 (Leica, Bensheim, Germany). The cell lysate was added, and the tube containing the micro-dissected islets was frozen with liquid nitrogen and stored at −80°C.

### Quantitative real-time PCR analysis

The total RNA was extracted using the RNeasy Micro Kit (Qiagen, Tokyo, Japan) according to the manufacturer's protocol. The reverse transcription reaction was carried out to synthesize first strand cDNA according to the protocols for the QuantiTect Reverse Transcription kit (Qiagen) at 42°C for 30 min (RT reaction) followed by 95°C for 3 min (deactivation of RTase). The synthesized cDNA samples were stored at −30°C.

Gene expression was analyzed using the BioMark 48.48 dynamic gene expression system (Fluidigm, South San Francisco, CA, USA). Preamplified cDNA was diluted with RNase-free water (1∶5) and used for quantitative PCR array analysis. A total of 33 genes were analyzed by the use of BioMark Real-Time PCR Analysis Software Version 2.0 (Fluidigm; Table S1). Actin, cytoplasmic 1 (Beta-actin) (*Actb*) was used as housekeeping gene.

### Statistical analyses

All data are expressed as the means ± SEM. Statistical significance was determined using Student's *t-*test, or a one- and two-factor analysis of variance (ANOVA). The Bonferroni correction was used as a post-hoc test when the data were determined to be significant by ANOVA. *P* values less than 0.05 was considered to be significant.

## Results

### The time-dependent changes of newly-formed vascular volume surrounding the islets

**Figure 2:**
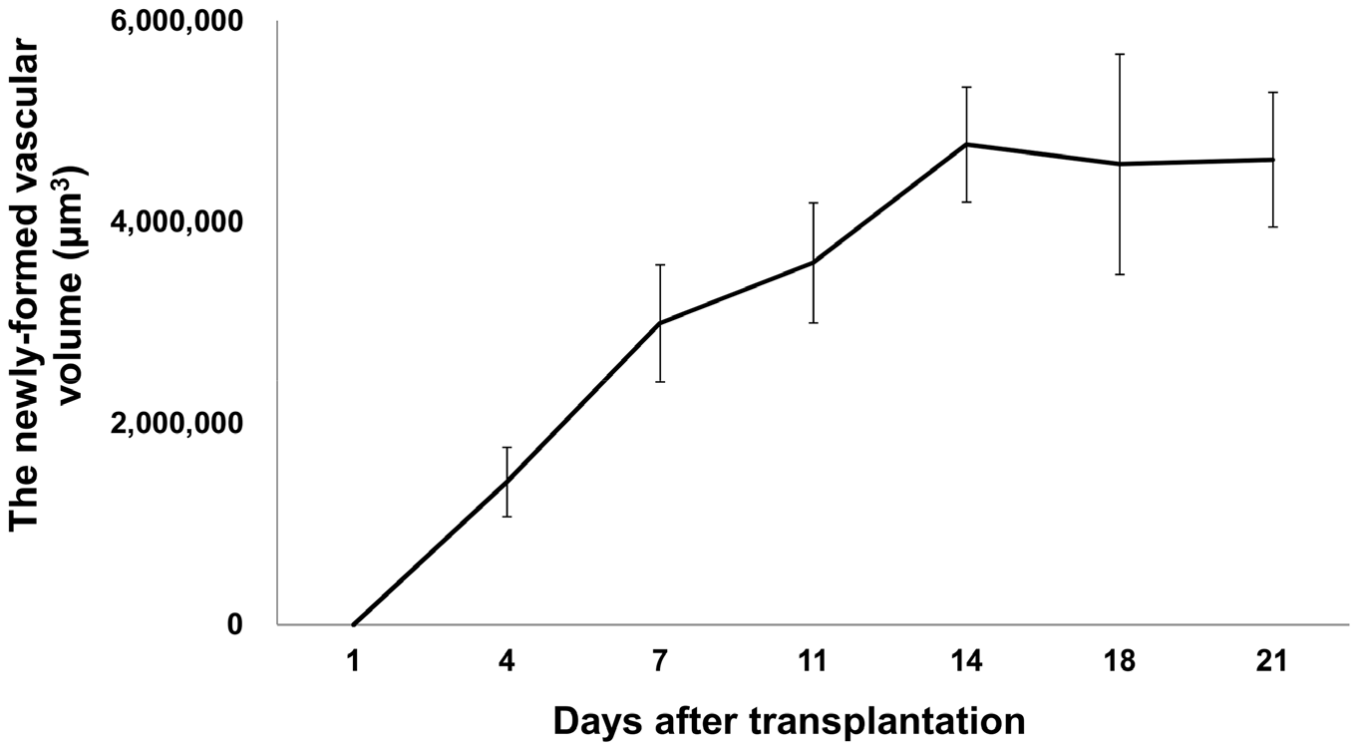
The time-dependent changes of newly-formed vascular volume for the transplanted pancreatic islets. Newly-formed vascular volume surrounding the islets at 4, 7, 11, 14, 18, and 21 days after transplantation was measured by using a Volocity system. All values are expressed as the means ± SEM.

**Figure 3:**
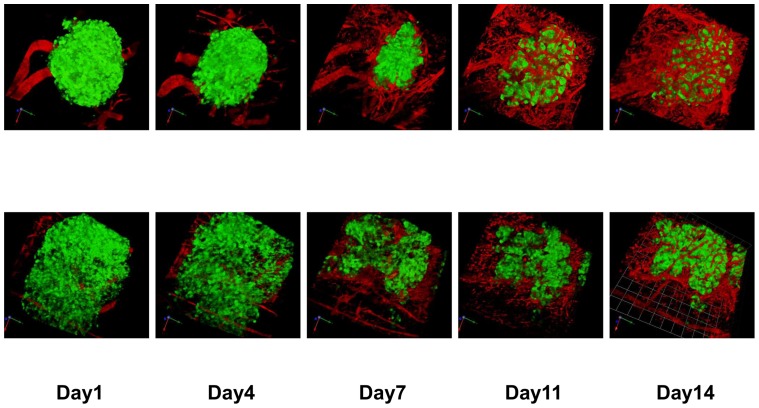
Imaging of the transplanted islets and surrounding blood vessels. Overlay of GFP fluorescence (pancreatic islets) and Texas Red fluorescence (blood vessels). Upper row: islets in the control group, Lower row: islets in the tacrolimus-treated group. GFP and Texas Red were excited at 890 nm. The scale bar indicated 51.0 μm/unit.

Capillary sprout formation made an appearance at 4 days after transplantation, and these sprouts were interconnected at day 7. The newly-formed microvascular network gradually got bigger. The newly-formed vascular volume surrounding the islet grafts increased until Day 14, and subsequently reached a growth plateau (Figs. 2 and 3).

### Tacrolimus concentrations in the whole blood

The blood concentrations of tacrolimus in the tacrolimus-treated group were 7.9±0.4 ng/mL (n = 6).

### Inhibition of islet revascularization by administration of tacrolimus

**Figure 4:**
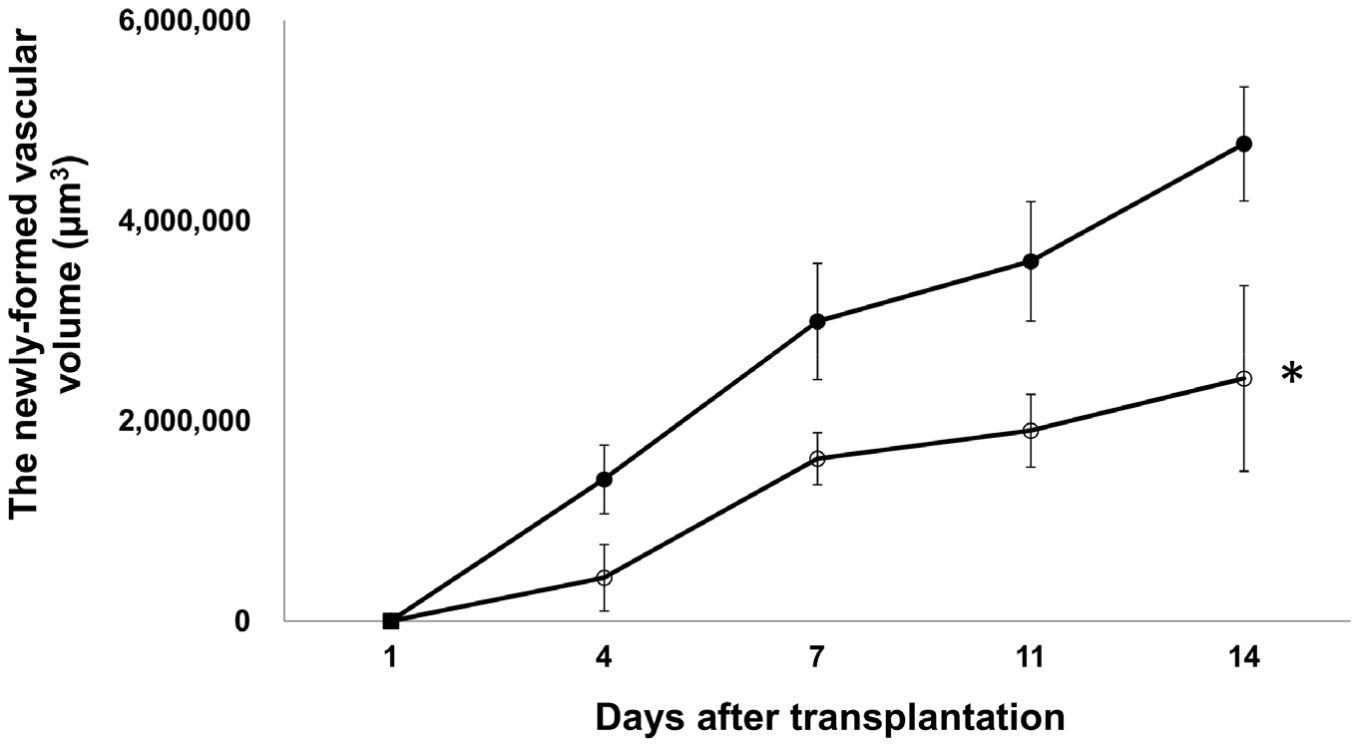
The time-dependent changes of newly-formed vascular volume surrounding the transplanted islets with and without tacrolimus. The newly-formed vascular volume in the control group (closed circles) and the tacrolimus-treated group (open circles). All values are expressed as the means ± SEM. *P<0.05 in comparison to the control group.

**Figure 5:**
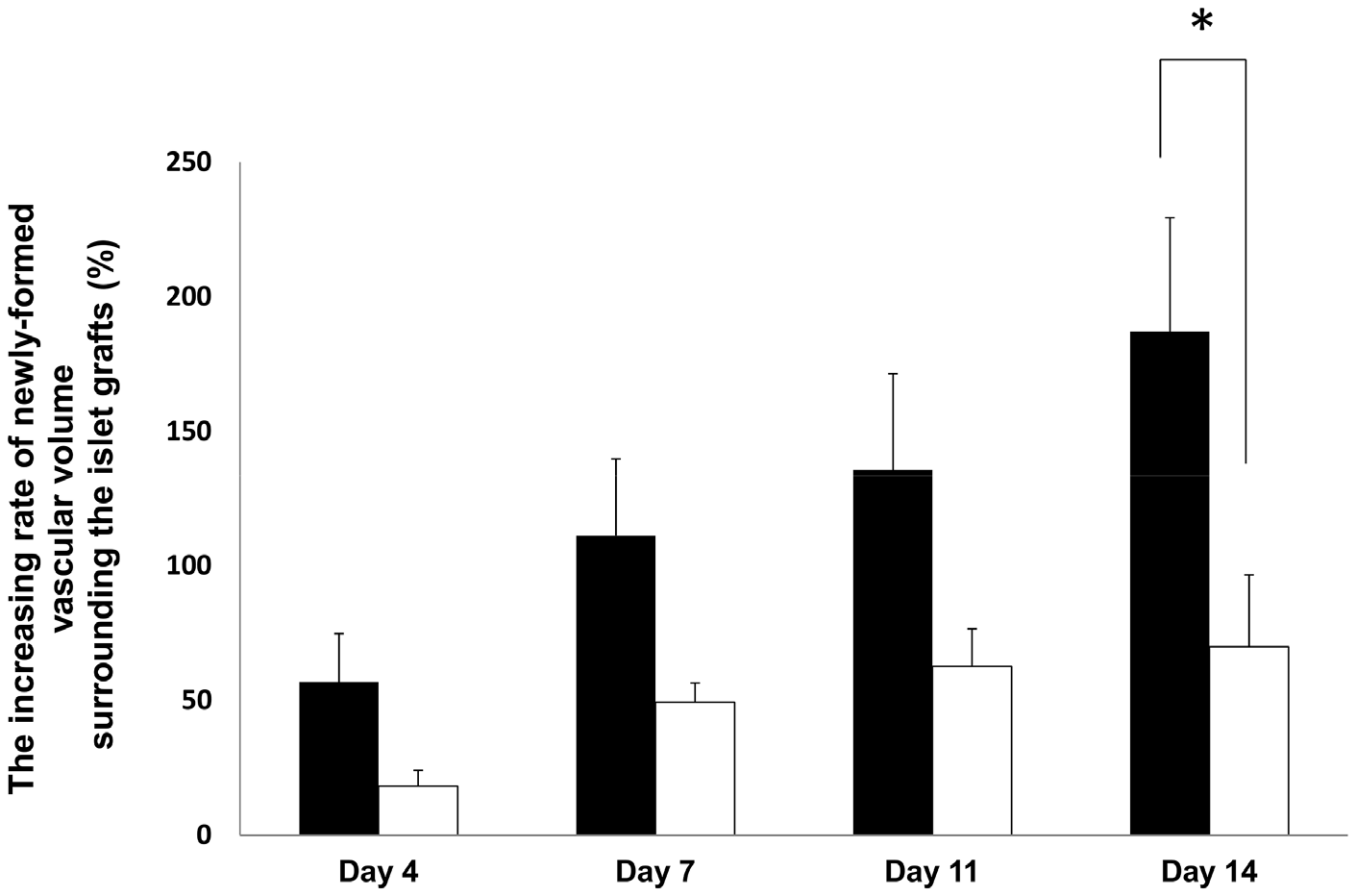
The increasing rate of newly-formed vascular volume surrounding the transplanted islets with and without tacrolimus. The increasing rate (against day 1) of newly-formed vascular volume surrounding the islets at 4, 7, 11, and 14 days after transplantation in the control group (black bar) and the tacrolimus-treated group (white bar) was calculated. All values are expressed as the means ± SEM. *P<0.05 in comparison to the control group.

The newly-formed vascular volume surrounding the transplanted islets in the tacrolimus-treated group was less than that in the control group (Fig. 3, p<0.05; Fig. 4). The increasing rate of newly-formed vascular volume surrounding the islets on day 14 was also significantly higher in the control group than in the tacrolimus-treated group (p<0.05; Fig. 5). The blood glucose levels in tacrolimus-treated group were maintained normoglycemic during the whole study period.

### The time-dependent changes of islet graft volume

**Figure 6:**
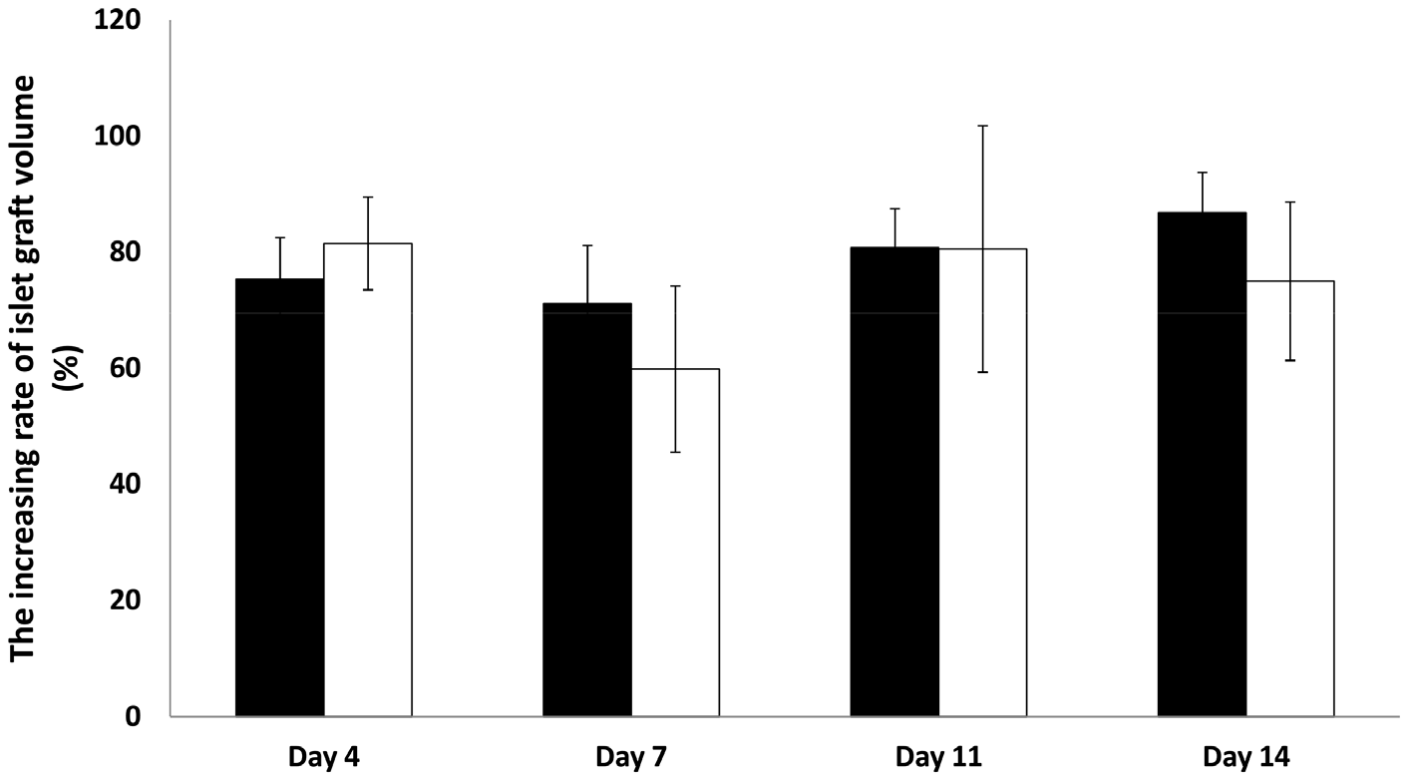
The increasing rate of islet graft volume with and without tacrolimus. The increasing rate (against day 1) of islet graft volume at 4, 7, 11, and 14 days after transplantation in the control group (black bar: n = 9) and the tacrolimus-treated group (white bar: n = 4) was calculated. All values are expressed as the means ± SEM.

The islet graft volume was almost identical throughout the whole study period in both groups (Fig. 6).

### Gene expression in the transplanted islets

**Figure 7:**
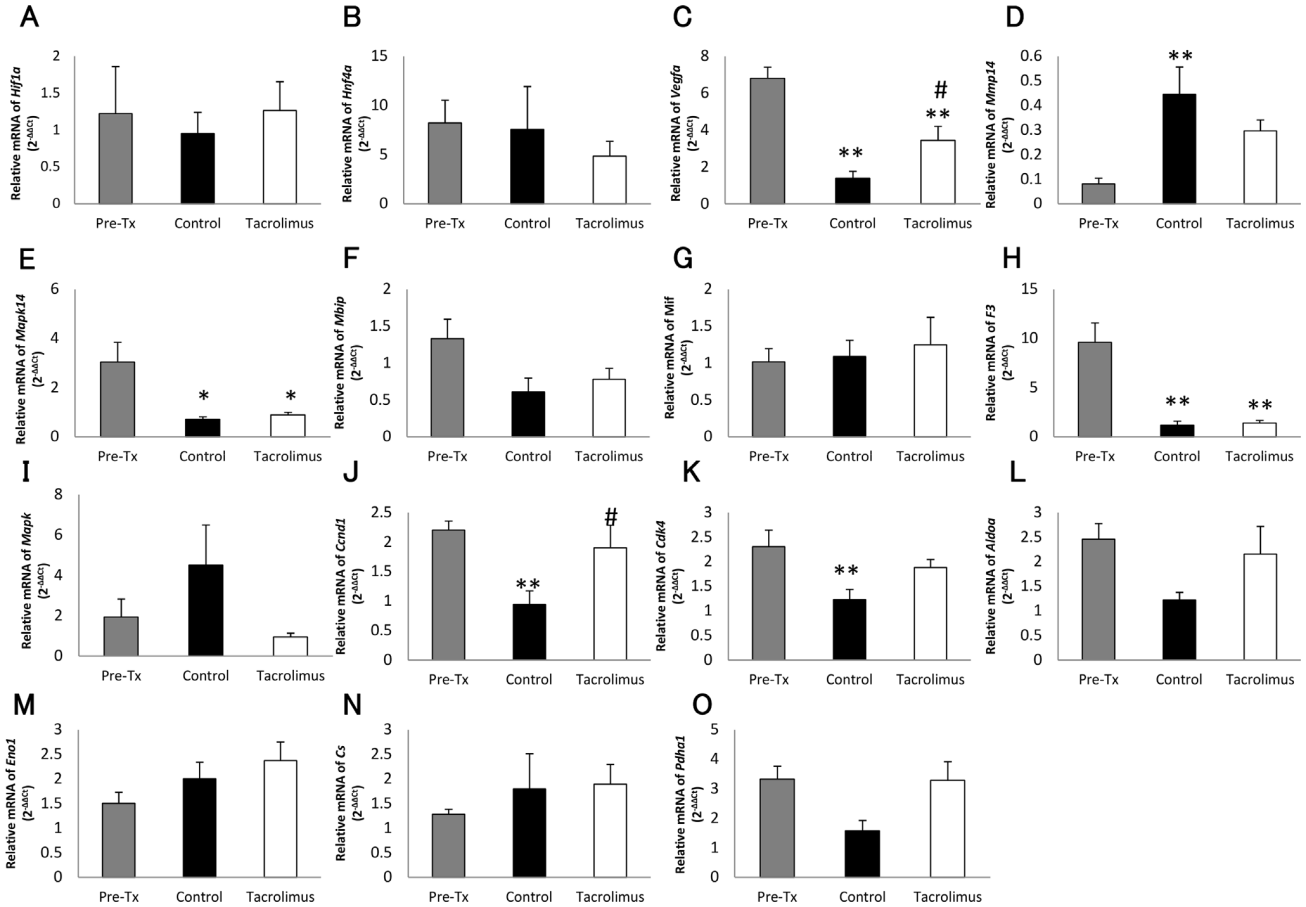
Relative gene expression in the transplanted pancreatic islets with and without tacrolimus. Relative gene expression of *Hif1a* (A), *Hnf4a* (B), *Vegfa* (C), *Mmp14* (D), *Mapk* (E), *Mbip* (F), *Mif* (G), *F3* (H),*Il1b* (I), *Ccnd1* (J), *Cdk4* (K), *AldoA* (L), *Eno1* (M), *Cs* (N), and *Pdha1* (O) in islets before transplantation (gray bar), the control group (black bar), and the tacrolimus-treated group (white bar). The values in the control and tacrolimus treated-group were represented as the ratio to the values in islets before transplantation. *P<0.05 in comparison to the islets before transplantation, **P<0.01 in comparison to the islets before transplantation, and #P<0.05 in comparison to the control group.

The expression of 33 genes related to inflammation, metabolism, cell cycle, and angiogenesis in the transplanted islets was analyzed. In comparison to the islets before transplantation, the expression of vascular endothelial growth factor A (*Vegfa*) (p<0.01), mitogen-activated protein kinase 14 (*Mapk14*) (p<0.05), tissue factor (*F3*) (p<0.01), G1/S-specific cyclin-D1 (*Ccnd1*), and cell division protein kinase 4 (*Cdk4*) (p<0.01) was suppressed in the transplanted islet grafts (control group) (Fig. 6). In contrast, the expression of matrix metalloproteinase-14 (*Mmp14*) (p<0.01) was upregulated in the transplanted islet grafts. Although the expression of *Vegfa* (p<0.05) and Ccnd1 (p<0.05) was significantly upregulated in the tacrolimus-treated group compared with that of the control group, no differences were observed between the tacrolimus-treated and control groups in terms of other types of gene expression (Fig. 7).

## Discussion

The outcome of islet allotransplantation is still far inferior to that of islet autotransplantation [2], despite the recent dramatic technical progress in the isolation of islets [3], [19], [20], [21]. Although several factors might contribute to this issue, the use of immunosuppressive drugs in islet allotransplantation could be one of the crucial factors. Tacrolimus is thought to have only marginal adverse effects on islet grafts, and consequently is used as the standard drug in both pancreatic islet and whole pancreas transplantation [22], [23]. Unlike whole pancreas transplantation, islet grafts are rendered avascular after enzymatic isolation and must become revascularized after pancreatic islet transplantation. However, the effects of tacrolimus on revascularization have been poorly studied. Therefore, the current study investigated the effect of tacrolimus on islet revascularization using a highly sensitive imaging system and found, for the first time, that tacrolimus substantially abrogates revascularization of transplanted islet grafts.

The present study introduced a highly sensitive imaging system by combining the DSC technique and MPLSM in order to observe the time-dependent changes of newly-formed vessels surrounding the islet grafts [11]. This system allowed the observation of the same islet grafts repetitively in identical recipient animals without histological sectioning. Moreover, the use of athymic mice as recipients allowed the revascularization process to be monitored for a longer observation period. This system demonstrated that the revascularization process is completed within 14 days after pancreatic islet transplantation. This finding is consistent with several previous reports [24], [25], [26], [27], [28], [29] performed by histological sectioning, thus indicating that this observation is accurate and also the established system was confirmed to be a useful tool to analyze the revascularization process of pancreatic islets.

This study further introduced the laser microdissection technique followed by real-time polymerase chain reaction (PCR) assay to analyze the state of gene expression in transplanted islet grafts *per se*. These combined methods using the highly sensitive imaging system and the laser microdissection technique together with real-time PCR assay allowed the simultaneous evaluation of the effect of immunosuppressive drugs not only on the revascularization process but also on the islet grafts *per se*. Notably, this elegant system could also be applicable for studying the angiogenesis and the engraftment in cancer cells and various stem cells.

The expression of *Vegfa*, *Mapk14*, *F3*, *Ccnd1* and *Cdk4* was suppressed in the islets transplanted without tacrolimus, in comparison to the islets prior to transplantation. However, unexpectedly, no difference was seen in hypoxia-inducible factor-1α (*Hif1a*) which is induced by hypoxia. These data suggest that the subcutaneous space might not be as hypoxic as expected. One possible explanation is that only revascularized islets survived and were observed in the present study. Another possible explanation is that only a few islets were transplanted into the DSC, and thus there were enough spaces for angiogenesis surrounding the islet grafts.

The islets transplanted with tacrolimus showed a significantly different expression of *Vegfa* and ccnd1 in comparison to the islets transplanted without tacrolimus, but no differences were observed between the tacrolimus-treated and control groups in terms of other gene expression. Since the expression of *Vegfa*, which is a major stimulator of revascularization by inducing proliferation and migration of endothelial cells and tube formation [30], [31], [32], was upregulated, it could be speculated that a stimulator of revascularization such as transforming growth factor-β1 and macrophage-colony stimulating factor was released from transplanted islets in response to inhibition of revascularization induced by tacrolimus. However, *Mmp14*, which promotes angiogenesis through extracellular matrix degradation and improves revascularization of transplanted islets [33], was not upregulated in the present study, although Deryugina et al. have reported that *Mmp14* expression can be associated with an upregulation of *Vegf* production [34]. A possible explanation for this discrepancy might be a transplant model, but the detailed mechanism remains uncertain. Therefore, further investigations on the relevance of other growth factors derived from donor islets such as hepatocyte growth factor (HGF) and basic fibroblast growth factor (bFGF) [35], [36] are also required in future studies. Considering that no suppression of analyzed gene expression and no reduction of islet graft volume were observed in the tacrolimus-treated group compared with the control group, the inhibitory effect of tacrolimus on islet revascularization may not be mediated by direct damage to the islet grafts. This also suggests a relatively lesser role for donor islet endothelial cells in revascularization process of islet grafts as shown by previous reports [27], [37]. Although rapamycin was reported to have deleterious effects on islet viability and growth factor-induced cell cycle regulation [38], [39], the present study suggests that tacrolimus appears to have no inhibitory effects on cell cycle regulation.

The dose of tacrolimus in the present study was determined according to previous studies. Lopez-Talavera et al. showed that the administration of tacrolimus at a dose of 0.5 mg/kg/day corresponds to a trough level of 6.0±1.5 ng/mL in rodents, which is similar to the dose used in the Edmonton protocol for humans [18]. Therefore, this dose was used for the tacrolimus-treated group. Islet revascularization was significantly inhibited after the administration of a clinical relevant dose of tacrolimus. Furthermore, Shapiro et al. reported that the portal C_max_ of immunosuppressive drugs was more than two times higher than the systemic C_max_ [40]. Therefore, the detrimental effect of tacrolimus on islet revascularization could be further enhanced when the islet grafts are transplanted into the liver, which is the current standard transplant site. As a result, the optimization of immunosuppressive drugs is of great importance for successful islet allotransplantation. FTY720, a sphingosine 1-phosphate analog, may be potentially useful and effective since previous studies have thus far shown it to effectively suppress immune responses in islet transplantation [41], [42]. The established new evaluation system in the present study could be a useful screening tool for optimizing the protocols of revascularization including the immunosuppressive regimen.

In summary, the present study demonstrated that tacrolimus inhibits the revascularization of isolated pancreatic islets without affecting the characteristics of the transplanted grafts. Further refinements in this immunosuppressive regimen, especially with regard to the revascularization of islet grafts, could therefore improve the outcome of islet allotransplantation.

## Supporting Information

Table S1 Genes analyzed for expression of transplanted islet grafts.
(DOCX)
