# Supplementary material for: Tacrolimus Inhibits the Revascularization of Isolated Pancreatic Islets
Source: PLoS One. 2013 Apr 17;8(4):e56799. doi: 10.1371/journal.pone.0056799 (PMC3629082; doi:10.1371/journal.pone.0056799)
Supplement: Supplementary file 1 [file pone.0056799.s001.docx]

gene symbol	gene name	Gene ID	Probe Number*	Left Primer	Right Primer
Notch1	Neurogenic locus notch homolog protein 1	ENSMUST00000028288.3	#80	ctggaccccatggacatc	aggatgactgcacacattgc
Hif1a	Hypoxia-inducible factor 1 alpha	ENSMUST00000021530.6	#95	gcactagacaaagttcacctgaga	cgctatccacatcaaagcaa
Aldoa	Fructose-bisphosphate aldolase A	ENSMUST00000032934.4	#97	tgccagtatgttactgagaaggtc	catgttgggcttcagcaa
Eno1	Alpha-enolase	ENSMUST00000080926.5	#77	gaggacactttcatcgcagac	ccagctcttcctcaattctga
Pdgfb	Platelet-derived growth factor subunit B	ENSMUST00000000500.6	#32	cggcctgtgactagaagtcc	gagcttgaggcgtcttgg
Trf	Serotransferrin	ENSMUST00000035158.7	#4	cataactatgtcactgccattcg	tcactggcgagttgtcgat
Serpine1	Plasminogen activator inhibitor 1	ENSMUST00000041388.3	#69	aggatcgaggtaaacgagagc	gcgggctgagatgacaaa
Mmp14	Matrix metalloproteinase-14	ENSMUST00000089688.3	#42	aacttcgtgttgcctgatga	tttgtgggtgaccctgactt
Ccnd1	G1/S-specific cyclin-D1	ENSMUST00000093962.3	#67	gagattgtgccatccatgc	ctcctcttcgcacttctgct
Cdk4	Cell division protein kinase 4	ENSMUST00000006911.4	#50	tcagtggtgccagagatgg	ggaaggcagagattcgctta
Vegfa	Vascular endothelial growth factor A	ENSMUST00000074629.4	#4	ttaaacgaacgtacttgcagatg	agaggtctggttcccgaaa
Col18a1	Collagen alpha-1	ENSMUST00000072755.4	#105	cagaaaggcagtgttggtga	cccaaggtctcctttgctc
Mbip	MAP3K12-binding inhibitory protein 1	ENSMUST00000021416.6	#50	tgcacaccctgacaagaca	aggctgggatgctgagag
Mif	Macrophage migration inhibitory factor	ENSMUST00000038169.5	#104	acagcatcggcaagatcg	ggccacacagcagcttact
Il6	Interleukin-6	ENSMUST00000026845.6	#6	tgatggatgctaccaaactgg	ttcatgtactccaggtagctatgg
Glp1	GATA-like protein 1	ENSMUST00000115494.1	#1	accttgtctggacaccgagt	ggcatgtaggccacagagac
Nkapl	NFKB activating protein-like	ENSMUST00000068235.3	#38	cacacctctcaagatgagaaacc	agccattgctgcaccttc
Gapdh	Glyceraldehyde-3-phosphate dehydrogenase	ENSMUST00000118875.1	80	tgtccgtcgtggatctgac	cctgcttcaccaccttcttg
Actb	Actin, cytoplasmic 1 (Beta-actin)	ENSMUST00000031564.4	56	aaggccaaccgtgaaaagat	gtggtacgaccagaggcatac
Ins1	Insulin-1	ENSMUST00000039652.3	#29	ttcagaccttggcgttgg	gtgcagcactgatccacaat
Gcg	Glucagon	ENSMUST00000102733.2	#33	acacagaggagaaccccaga	gtggcgtttgtcttcattca
Sst	Somatostatin	ENSMUST00000004480.2	#53	cccagactccgtcagtttct	gggcatcattctctgtctgg
Pdx1	Pancreas/duodenum homeobox protein 1	ENSMUST00000085591.3	#51	gaaatccaccaaagctcacg	cgggttccgctgtgtaag
F3	Tissue factor	ENSMUST00000029771.7	#38	tccaggaaaactaaccaaaatagc	tcccacaatgatgagtgtttct
Cs	Citrate synthase	ENSMUST00000005826.6	#100	ggaaggctaagaacccttgg	tcatctccgtcatgccatagt
Pdha1	Pyruvate dehydrogenase E1 component subunit a	ENSMUST00000033662.7	#40	gtcacttgtgtgatggtcagg	gtggtccgtagggtttatgc
Pdk1	[Pyruvate dehydrogenase [lipoamide]] kinase iso	ENSMUST00000006669.4	#20	gttgaaacgtcccgtgct	gcgtgatatgggcaatcc
Hnf4a	Hepatocyte nuclear factor 4-alpha	ENSMUST00000109411.1	#27	cagcaatggacagatgtgtga	tggtgatggctgtggagtc
Vegfb	Vascular endothelial growth factor B Precursor	ENSMUST00000025914.6	#216	gctcaacccagacacctgtag	aggaggttcgcctgtgct
Mapk14	Mitogen-activated protein kinase 14	ENSMUST00000114752.1	#227	gaccttctcatagatgagtggaaga	caggactccatttcttcttggt
Il1b	Interleukin-1 beta Precursor	ENSMUST00000028881.6	#215	tgtaatgaaagacggcacacc	tcttctttgggtattgcttgg
Pik3r1	Phosphatidylinositol 3-kinase regulatory subunit a	ENSMUST00000035532.5	#36	gacggcactttccttgtcc	tgacttcgccgtctaccac
Ccne1	G1/S-specific cyclin-E1	ENSMUST00000108023.2	#82	ctgagagatgagcactttctgc	gagcttatagacttcgcacacct

Supplementary Table. Genes analyzed for expression of transplanted islet grafts

*Roche universal probe library
